# ‘maskBAD’ – a package to detect and remove Affymetrix probes with binding affinity differences

**DOI:** 10.1186/1471-2105-13-56

**Published:** 2012-04-16

**Authors:** Michael Dannemann, Michael Lachmann, Anna Lorenc

**Affiliations:** 1 Max Planck Institute for Evolutionary Biology, August–Thienemann–Str. 2, 24306, Plön, Germany; 2 Max Planck Institute for Evolutionary Anthropology, Deutscher Platz 6, 04103, Leipzig, Germany

## Abstract

**Background:**

Hybridization differences caused by target sequence differences can be a confounding factor in analyzing gene expression on microarrays, lead to false positives and reduce power to detect real expression differences. We prepared an R Bioconductor compatible package to detect, characterize and remove such probes in Affymetrix 3’IVT and exon-based arrays on the basis of correlation of signal intensities from probes within probe sets.

**Results:**

Using completely mouse genomes we determined type 1 (false negatives) and type 2 (false positives) errors with high accuracy and we show that our method routinely outperforms previous methods. When detecting 76.2% of known SNP/indels in mouse expression data, we obtain at most 5.5% false positives. At the same level of false positives, best previous method detected 72.6%. We also show that probes with differing binding affinity both hinder differential expression detection and introduce artifacts in cancer-healthy tissue comparison.

**Conclusions:**

Detection and removal of such probes should be a routine step in Affymetrix data preprocessing. We prepared a user friendly R package, compatible with Bioconductor, that allows the filtering and improving of data from Affymetrix microarrays experiments.

## Background

In microarray gene expression analysis, there is an assumption that a probe has the same set of targets in compared groups and therefore differences in probe signal intensity are caused by different levels of gene expression. This assumption does not hold when experimental groups with different genetic makeup are compared, as the target region of a specific probe may contain SNPs and other sequence differences. Experimental groups may also differ in their set of expressed isoforms or cross-hybridizing targets. The latter issue may arise even between genetically identical samples, such as when comparing different tissues, or samples differing in applied treatment. The binding affinity for such probes will differ between exprimental groups and the difference in signal intensity will be confounded with transcript abundance. We termed probes with such binding affinity differences BAD probes.

BAD probes lead to errors in estimates of differential gene expression [1], disruption of eQTL mapping [2,3] and errors in resolving cis and trans effects [4]. We ourselves have shown that BAD probes both introduce spurious gene expression differences and, by disrupting normalization, reduce the power to detect true ones [5]. On the other hand, BAD probes might be useful as genetic markers distinguishing species or strains (called also single feature polymorphisms, SFPs) [6,7].

The number of affected probes depends on the genetic distance between experimental groups. Even with 1% nucleotide differences, as observed between human and chimpanzee or Mus musculus and Mus spretus, SFPs due to SNPs alone will appear in ~22% of 25-nucleotide probes.

To overcome this problem, several approaches were employed, including: removal of probes overlapping known polymorphisms [8-10], adding group as a factor in probe-level analysis [1,11] and methods based on correlating expression values within a probe set [6,7,12,13]. The advantage of the last approach is that information concerning sequences or actual targets (isoform and cross hybridizing targets) need not be known. It also alows one to discover SFPs.

**Figure 1:**
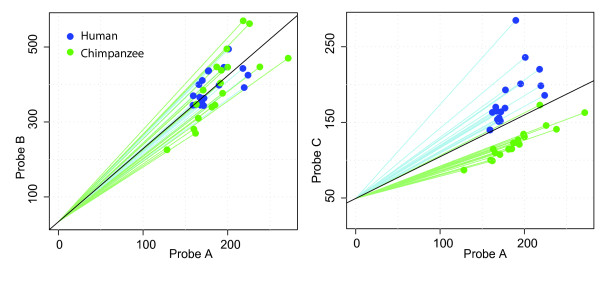
**Principle of the masking method.** Each dot represents a sample— human or chimpanzee. On the axes are fluorescence intensities for the probes from the same probe set. Our method performs a t-test of the slopes to each point (green and blue lines), assuming that the intercept is taken from all points (black line). On the left ( **a**) there is no binding afinity difference between humans and chimpanzees in either probe. On the right ( **b**) one of the probes is BAD.

Previously, we proposed a method to detect BAD probes on Affymetrix gene expression microarrays based on the expression values themselves, as we described in [5]. Briefly, the signal for a given probe is proportional to the amount of RNA in the sample and its binding affinity. When one target is measured with several probes (a probe set), as on Affymetrix arrays, the probe signals are correlated for each sample. BAD probes correlation differs between groups, hence comparison of pairwise correlations between those groups allows to identify of BAD probes, as summarized on Figure1.

We present an R package implementing this method for the detection and removal of BAD probes from the Affymetrix gene expression data. Functions in this package improve upon the version that we made available with the original paper [5], are compatible with current R Bioconductor framework, and work for both Affymetrix 3’IVT and exon-based arrays. The package with a vignette and example data was submitted to Bioconductor. We re-validated the method with recently available full genome data for mouse strains and compared its performance with competing software. We also employed masking on a human tumour-healthy tissue data set, to test for presence of BAD probes between tissue types and their influence on differential gene expression detection. In such comparisons, the difference in targets between experimental groups and impact of BAD probes has been overlooked to date.

## Implementation

### BAD probes detection

We implemented the method described in [5]. Briefly, a signal for the probe j on the array i might be expressed as a sum of baseline response due to nonspecific hybridization (*ν*_*j*_), abundance of target RNA (*Φ*_*i*_) multiplied by binding strength of probe j (*θ*_*j*_) and an error term.

If probes *O*_*i1*_ and *O*_*i2*_ hybridize to the same transcript, the relationship between their fluorescence levels might be expressed as

(1) $$O_{i2}=\frac{v_{2}\theta_{2}-v_{1}\theta_{2}}{\theta_{1}}+\frac{\theta_{2}}{\theta_{1}}O_{i1}+\in$$

When samples differ between groups in binding affinities and/or background intensity, this linear relationship still holds within each group, but their slopes differ between groups.

We test the null hypothesis that in both groups the binding strength, as well as the background binding level, are the same,

1. We estimate the intercept for combined groups

(2) $$\beta =\frac{v_{2}\theta_{1}-v_{1}\theta_{2}}{\theta_{1}}$$

2. We test with t-test if distribution of *(O*_*i2*_* − β)/O*_*i1*_ is the same for both groups and record its P-value.

(3) $$\frac{O_{i2}-\beta }{O_{i1}}=\frac{\theta_{2}}{\theta_{1}}+\in$$

3. The same test (1-2) is performed for all probe pairs, in both directions. We thus build a *J* ×  *J* matrix of all pairwise tests, less the diagonal.

4. For each probe in a probe set we calculate the geometric mean of all *P*-values in the matrix where this probe is involved. We record the probe with the smallest geometric mean of *P*-values (mP-value) and exclude this probe’s comparisons from the matrix.

5. The previous step is repeated, until the matrix contains only two probes – they are both assigned geometric mean of their pvalues.

Then a cutoff is chosen and all probes with an mP-value below this cutoff are designated as BAD.

### Description of the package

The package allows detection of BAD probes in Affymetrix microarrays designed with several probes measuring a target, 3’ IVT gene expression arrays and whole–transcript expression arrays (exon and gene arrays). Standard Bioconductor gene expression data structures are supported, thus the package may be easily be used with other Bioconductor tools.

As input for detection of BAD probes the package requires raw expression data in the form of an AffyBatch object and a group assignment of all samples. Detection of BAD probes in expression data works only for probe sets targeting transcripts that are expressed – others, picking background and crosshybridization signal are meaningless. Therefore by default, probe sets are evaluated with mas5calls() function from the package affy and only probe sets which are expressed (“P” call) in at least 90% of samples in each group are retained for BAD probes detection. However, the user may choose to analyze a custom set of probe sets or use average probes values to define probe sets to analyze. The masking achieves sufficient power with at least five samples in each group to compare (groups may differ in size). The resulting list contains a mask - a data frame with quality scores for all analyzed probes.

A low quality score means that a probe is likely to be BAD and probes with quality value below a certain cutoff may be removed from expressionSet and downstream analysis. Ranking of probes by quality scores depends on the data set (sample size and number of probes within probe sets) and hence a threshold for excluding probes from downstream analysis is data set specific. The package contains several functions to assist with choice of cutoff score. A stringent (high) cutoff might be used to compare gene expression between groups, whereas a relaxed (low) cutoff may be used to identify candidate SFPs, because then a low rate of false positives is important.

When BAD status is known for a subset of probes, a cutoff might be chosen to match desired sensitivity and specificity level, on the basis of this subset analysis. Function overlapExpExtMasks() computes type 1 and type 2 errors and its confidence intervals for different cutoffs and then plots them. Also, the distributions of quality scores for probes defined as BAD and non-BAD might be compared using a Kolmogorov-Smirnov or Wilcoxon test, to estimate if cutoff is too stringent and prevents discrimnation of BAD and non-BAD probes.

In the absence of a reference subset of probes, distribution of quality scores might suggest an appropriate cutoff level.

It is advisable to inspect some probes that have borderline quality score. The function plotProbe() allows comparison of a probe’s intensities against all other probes from the same probe set.

Probes may be removed from the original AffyBatch object with prepareMaskedAffybatch(). It returns a new affyBatch object and an appropriate CDF environment, which are both devoid of BAD probes. At this step the user may decide what is a minimal number of probes left to still keep a probe set in the analysis. For prepareMaskedAffybatch() BAD probes may be identified by a mask object and a cutoff value or as a list of probes to remove.

After removal of BAD probes, expression estimates might be obtained in any way preferred by the user, as for every probe set the remaining probes are treated as a redefined probe set. Masking might be coupled also with probe set redefinition by a custom cdf.

The package is compatible with 3’ IVT arrays and gene/exon arrays. The resulting affyBatch object may be used downstream as a usual affyBatch object.

### Performance

We took the opportunity to use the recent sequencing of 20 inbred mouse strains as a comprehensive source of information about SNPs and indels. We detected BAD probes in a public dataset of striatum expression from C57BL/6NJ and DBA/2 J strains [[14], GEO Series GSE26024], measured on the MOE430 2.0 array based on C57BL sequences. We estimated false positives (probes detected as BAD with no polymorphism) and false negatives (undetected BAD probes) of expression-based mask by comparison with polymorphisms in probe target regions in a similar way as in [5].We did the same with SNEP [15], which is a method to detect BAD probes shown to be superior to several other approaches and not compared with ours before.

We also tested for the presence of BAD probes and masking effects in a public dataset comprising 17 healthy tissue and 20 tumour samples from human lung ([16], data from http://www.broadinstitute.org/mpr/lung/), measured on U95 arrays. The mask was produced with default features and a cutoff of 0.001 (1373 probes masked – 4.5%), determined from the distribution of probes’ quality scores. Probe sets were defined as being differentially expressed if, after gcrma normalization, their Benjamin-Hochberg corrected t-test p-values were smaller than 0.01.

## Results and Discussion

**Figure 2:**
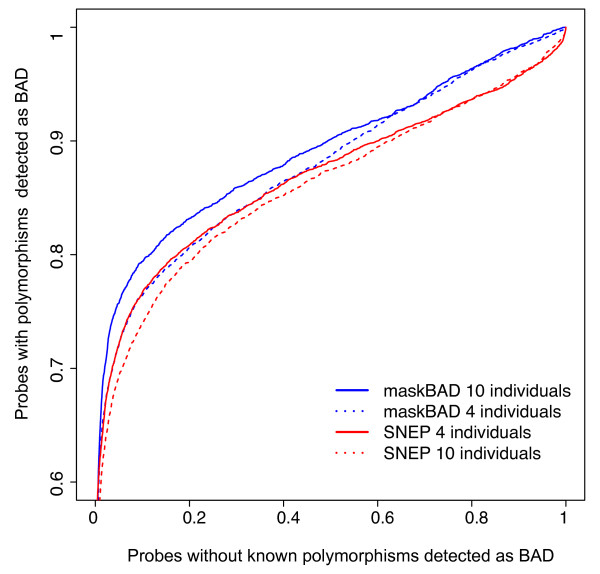
**Detection of known polymorphisms by maskBAD.** Performance maskBAD compared with SNEP algorithm for the mouse dataset. X-axis: fraction of probes masked, but without known polymorphism. Y-axis: detected fraction of known SNPs/indels. Masks were build using 4 or 10 individuals from each group.

The package maskBAD is designed to detect and remove BAD probes and the resulting bias in expression estimates. Previously we demonstrated the high accuracy and detection rate of the method in detecting artificially introduced BAD probes [5]. We detected 90% of probes with an artificially introduced difference and masked only 1.8% of probes without a difference. We estimated also false positive and false negative rates in actual data from two mouse strains and a human-chimpanzee comparison. However, this approach suffered from incomplete mouse polymorphism data at the time, and unknown polymorphisms between human and chimpanzee individuals used in the expression studies. However, with the full genome information for inbred mouse strains it was possible to estimate it with greater accuracy [Figure2. According to the sequence data, within the 22748 expressed probe sets, 2.4% of probes were affected by either a SNP or an indel. At cutoff 0.032 we detect 76.2% of known SNPs/indels, at the same time masking only 5.5% of probes without any known polymorphism. Still, some of the probes without known polymorphisms might differ in binding affinity because of other differences between experimental groups, for example in additional file 1 we show a probe (quality score = 2.46e − 10) without a known polymorphism between mouse strains and with obvious BAD behavior.

We compared the performance of our method with SNEP [15] using the same dataset. For group sizes 4 and 10 our method is equally good for low cutoffs and superior for high cutoffs [Figure2. When detecting 75.6% of real SNPs/indels, it detects as BAD 8.5% probes without SNP/indel and with type 2 error 5.5% it detects 72.8% of known SNPs/indels.

We find also that BAD probes are present in the cancer-healthy tissue comparison and influence DE detection. We analyzed 1913 (15%) probe sets which were expressed in both groups and identified 1373 (4.5%) BAD probes at cutoff 0.001 between carcinoid lung tumors and healthy lung tissue. Such differences between two human tissues mean qualitative differences in targets, such as cross-hybridizing targets or isoforms between tumour and normal tissue. We enquired how masking influences differential expression detection. There were 656 probe sets detected as differentially expressed (DE) both before and after masking, while 94 probe sets were only DE before masking and 38 were only DE after masking.

A single BAD probe can cause an extreme difference in calling differential expression. For example, a probe set (38657_s_at), targeting clathrin, before masking showed no difference in expression (p = 0.41). By removing probe 3 (detected as BAD: quality score = 2.5e-5, additional file 2), the P-value becomes highly significant (p = 0.009). The probe response shows the opposite direction of intensity change for this probe than the others, leading to a biased expression estimate.

On the other hand, in probe set 37766_s_at differential expression detected without masking (p = 0.01) is an artifact of only one BAD probe (x = 420 y = 307, quality score = 5.5e-6) in this probe set ( Additional file 3) and disappears after masking (p = 0.08). This detected difference might be still of interest, as it might indicate differential isoform expression or RNA editing between two tissues, or identical somatic mutations in tumour samples, but it is not a “simple” difference in expression levels.

**Figure 3:**
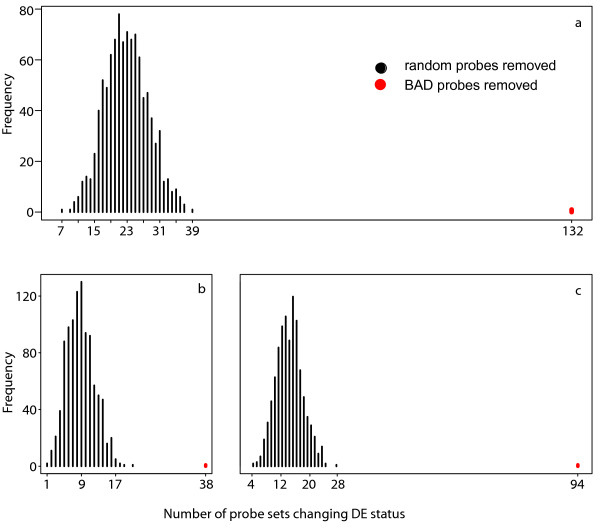
**Probe sets changing DE status after removal of BAD or random probes.** Number of probe sets changing DE status after removal of 1373 probes, randomly chosen (sampled 1000 times) or identified as BAD probes. ( **a**) All probe sets ( **b**) probe sets detected as DE only after masking, ( **c**) probe sets loosing DE status after masking.

If removal of BAD probes reveals a real biological signal, and does not not just introduce a random change in expression estimates due to a reduction in the number of probes in probe set, than when discarding BAD probes one should see much a bigger difference in DE estimates compared to removing probes randomly. To test this, we ran a simulation 1000 times where we removed the same number of probes, with the same distribution of probes per probe set, as the set of probes we removed as BAD. The number of probe sets detected as DE was much smaller (Figure3).

As we have shown previously [5], excluding BAD probes both removes artifacts and reveals a real biological signal. Previously, it was used in comparisons between genetically different strains or species [12,17-21]. Systematic differences in probes’ targets might also be present between different tissues (as in the previous cancer-healthy tissue comparison) or samples subjected to different treatments. Whereas regions of known differences might be accommodated in array design (by careful design and annotation of separate probe sets targeting known isoforms and exon-level analysis), other differences that are not recognized might lead to errors in detecting DE. To date, their identification and removal is usually not a part of a 3’IVT arrays data analysis pipeline. As they differ in binding affinity between compared groups, but are still in relation to other probes within a probe set, downweighting of outlier probes, as performed in summarization step of preprocessing methods (RMA, GCRMA, affyILM, plier etc.) does not reduce their interference.

BAD probes violate the assumption made in methods for estimating gene expression from microarrays, that there is a common binding affinity for a given probe for all samples. Therefore, for DE gene discovery, they should be removed from analysis.

As BAD probes are a result of systematic differences between compared groups, they might identify candidate loci for further investigation, for example differing in splicing isoforms between compared groups. When using expression data to classify samples, they might be another factor discriminating samples. However, as they obscure real expression levels, by potentially canceling out expression differences with reciprocal signal or by disrupting the normalization process, they should be removed before estimating expression level differences.

Therefore BAD probe detection should be a part of standard differential gene expression analysis. When a goal is to find both quantitative and qualitative differences in expression between samples, the analysis might be done twice, with and without BAD probes. Comparison of results for the same dataset before and after masking, along with the localization of BAD probes, would help to interpret which probe sets apparently detecting DE differ in expression levels and which have rather qualitative target differences.

A set of probes identified as BAD for a specific experiment might be removed from analysis of other similar datasets. However, each tissue, developmental stage etc. might differ qualitatively in transcriptome and therefore have a different set of BAD probes, so identification of ones specific for the experiment is better.

## Conclusions

We introduce “maskBAD”, the R package to detect and remove probes with different binding affinity in Affymetrix array expression data. The method implemented in maskBAD performs better than other methods in detecting BAD probes. Identification and removal of BAD probes removes spurious gene expression differences and helps to reveal real ones. In clustering analysis of gene expression, identification of BAD probes guides interpretation of discriminating probe sets.

The BAD probes are prevalent in comparisons of genetically distinct samples, such as different strains of a species or between species, but systematic qualitative differences in transcriptome might introduce them also when samples differ by treatment, health status or tissue type. All commonly used preprocessing procedures assume constant binding affinity for a probe in all samples and their downweighting of outlier probes does not protect from BAD probes. Therefore masking should be a routine step in data preprocessing. The “maskBAD” package allows identifying, inspection and removing BAD probes in R and Bioconductor environment and make it a part of standard gene expression analysis pipeline.

## Availability and requirements

Project name: Masking BAD microarray probes.

Project home page: http://bioinf.eva.mpg.de/masking/test/pmwiki.php/Site/MaskingMicroarraysProbesWithBindingAffinityDifferences and Bioconductor (http://bioconductor.org/packages/2.10/bioc/html/maskBAD.html)

Operating systems: Linux, OS X, Windows

Programming language: R (currently requires R-devel version)

Other requirements: packages affy, gcrma

License: GNU GPL

## Links

Package is available at

http://bioconductor.org/packages/2.10/bioc/html/maskBAD.html

http://bioinf.eva.mpg.de/masking/test/pmwiki.php/Site/MaskingMicroarraysProbesWithBindingAffinity Differences

## Abbreviations

BAD, binding affinity different; DE, differential expression; SFP, single feature polymorphism; SNP, single nucleotide polymorphism.

## Misc

Michael Dannemann and Anna Lorenc contributed equally.

## Competing interests

The authors declare that they have no competing interests.

## Authors' contribution

AL, MD and ML conceived the package. MD developed the package. MD and AL tested the package. AL wrote the manuscript, all authors have contributed to it and have read and accepted the final version.

## Supplementary Material

Additional file 1 **A BAD probe without known polymorphisms in the targets.** Examples of fluorescence intensities for probes from the probe set 1415723_at, without any polymorphisms in target region according to sequence data, for mice from C57BL/6NJ (green) and DBA/ 2 J (blue) strains. A. BAD probe (quality score 2.46e - 10) B. Probe without BAD (quality score 0.876). Click here for file

Additional file 2 **BAD probe introduces spurious DE.** Fluorescence levels of the probe set 37766_s_at for normal lung and tumor lung tissue. Each dot represents a sample. This probe set is detected as DE (alpha = 0.01) with raw data, but is not significant after masking. Intensities and its correlations with some other probes of the probe set are shown for the probe 15, identified as BAD (left) and probe 13, non-BAD (right). In the middle fluorescence levels for consecutive probes. Click here for file

Additional file 3 **BAD probe prevents detection of DE.** Fluorescence levels of the probe set 38657_s_at for normal lung and tumor lung tissue. Each dot represents a sample. This probe set is detected as DE (alpha = 0.01) only after masking. Intensities and its correlations with some other probes of the probe set are shown for the probe 3, identified as BAD (left) and probe 4, non-BAD (right). In the middle fluorescence levels for consecutive probes. Click here for file
