# Supplementary figures and images for: ‘maskBAD’ – a package to detect and remove Affymetrix probes with binding affinity differences
Source: BMC Bioinformatics. 2012 Apr 16;13:56. doi: 10.1186/1471-2105-13-56 (PMC3439685; doi:10.1186/1471-2105-13-56)

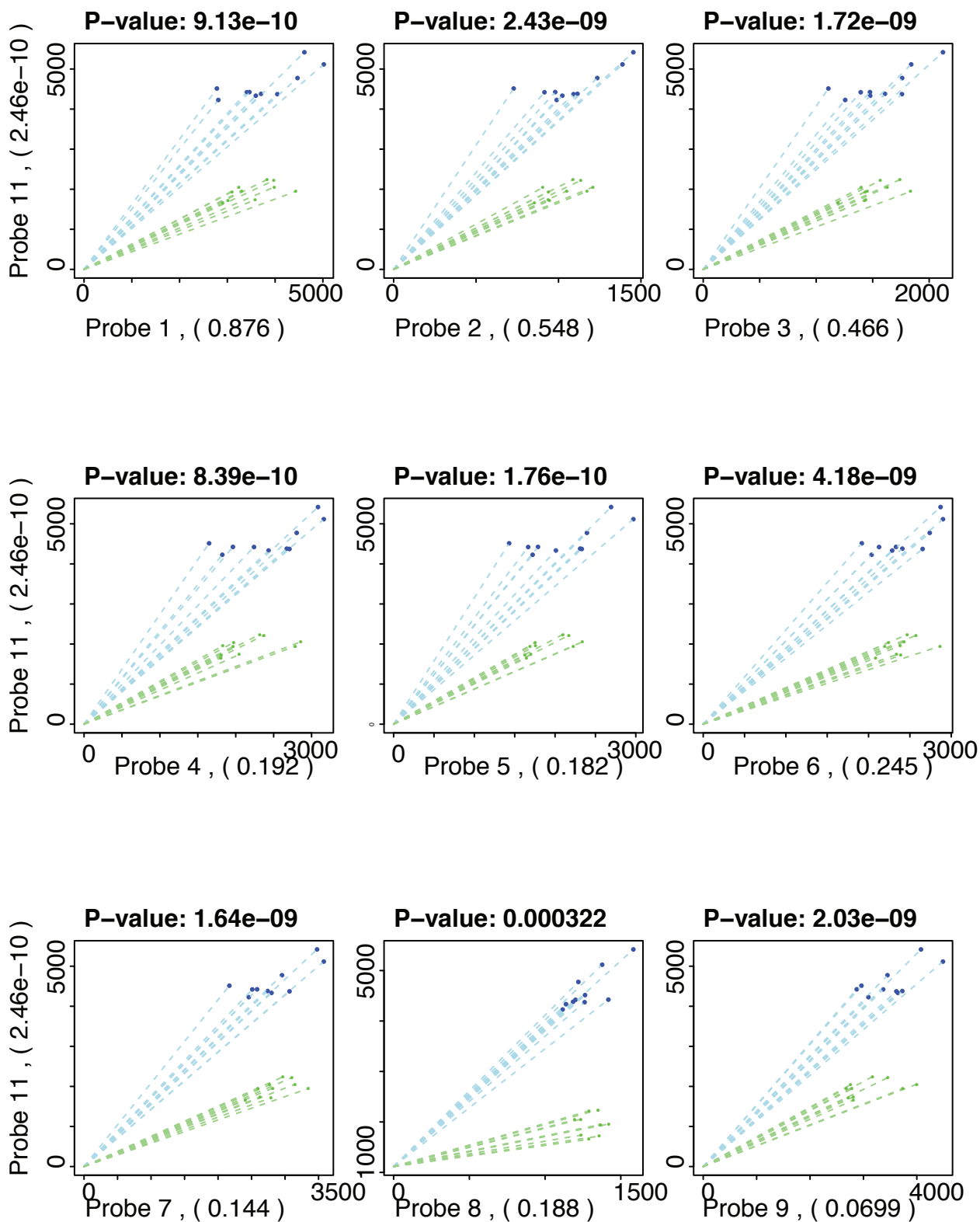

Samples from strains:

DBA/2J

C57BL/6NJ

Supplement: Additional file 1 — A BAD probe without known polymorphisms in the targets. Examples of fluorescence intensities for probes from the probe set 1415723_at, without any polymorphisms in target region according to sequence data, for mice from C57BL/6NJ (green) and DBA/ 2 J (blue) strains. A. BAD probe (quality score 2.46e - 10) B. Probe without BAD (quality score 0.876). [file 1471-2105-13-56-S1.pdf]

Probe 15 - quality score  $4.5e-06$

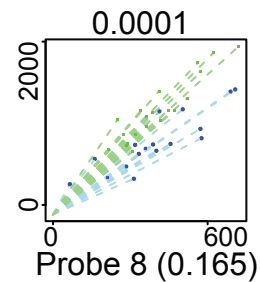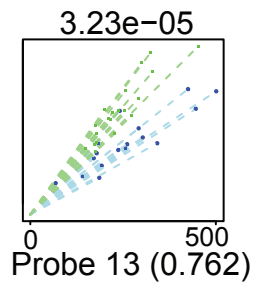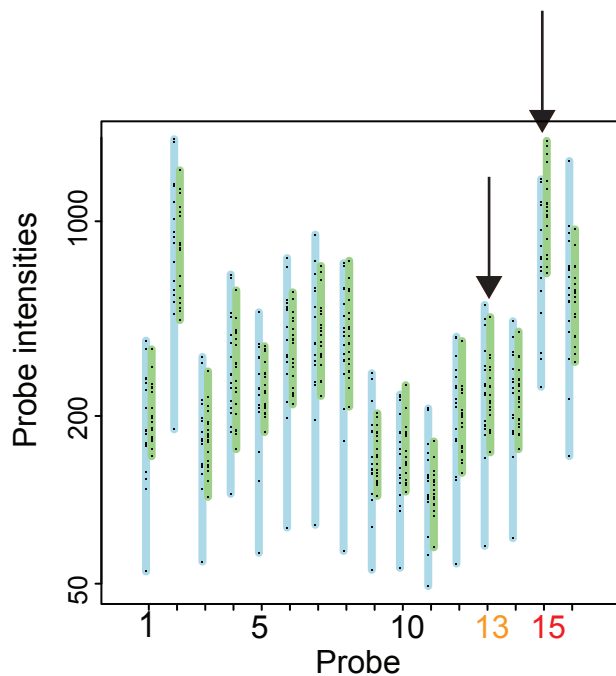

Probe 13 - quality score 0.76

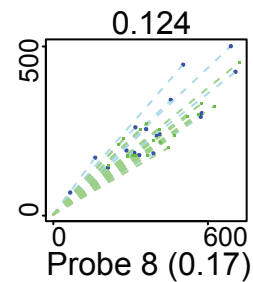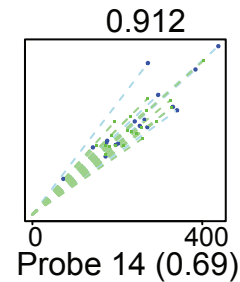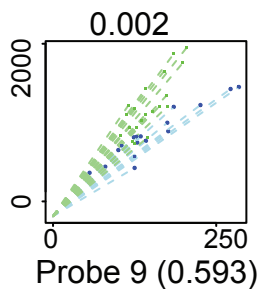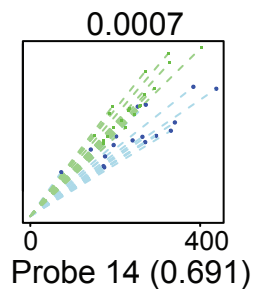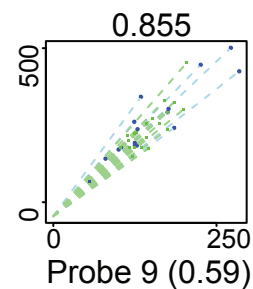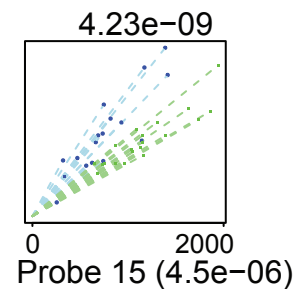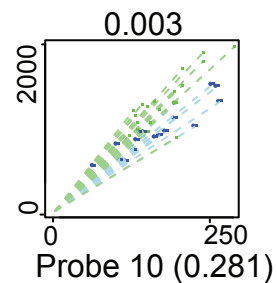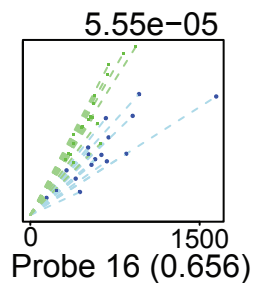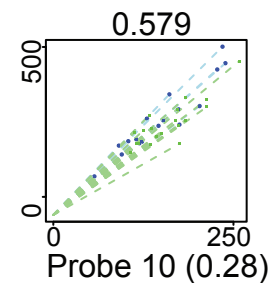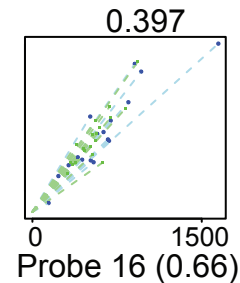

Supplement: Additional file 2 — BAD probe introduces spurious DE. Fluorescence levels of the probe set 37766_s_at for normal lung and tumor lung tissue. Each dot represents a sample. This probe set is detected as DE (alpha = 0.01) with raw data, but is not significant after masking. Intensities and its correlations with some other probes of the probe set are shown for the probe 15, identified as BAD (left) and probe 13, non-BAD (right). In the middle fluorescence levels for consecutive probes. [file 1471-2105-13-56-S2.pdf]

Probe 3

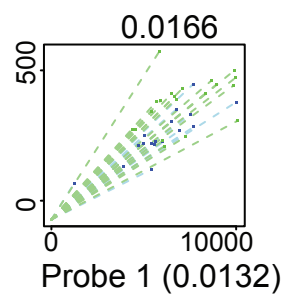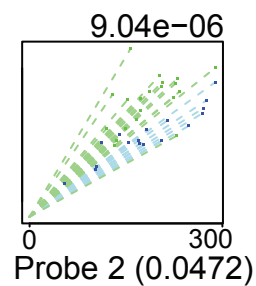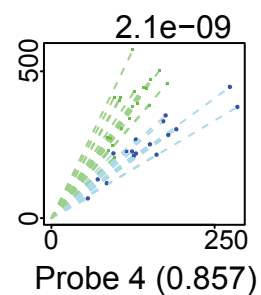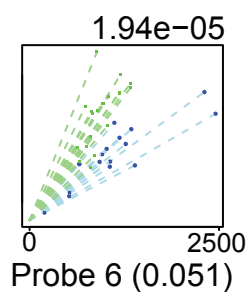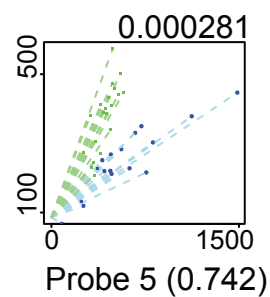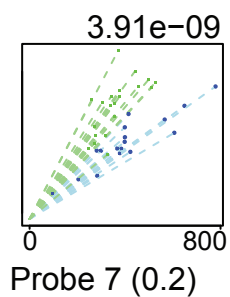

Probe 4

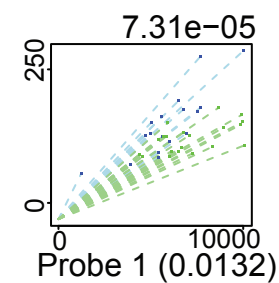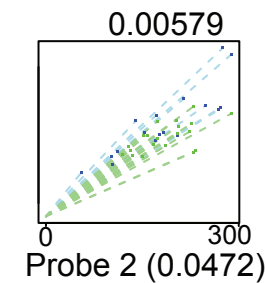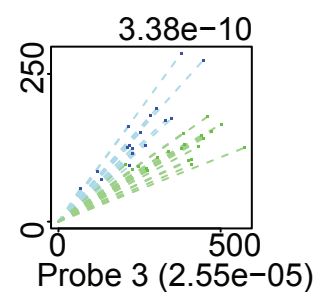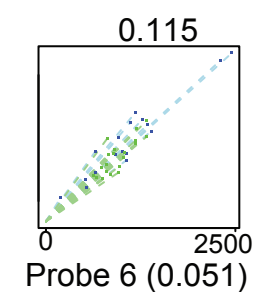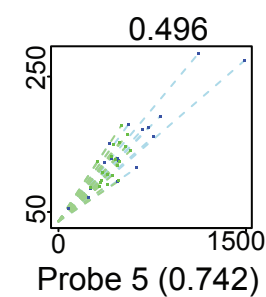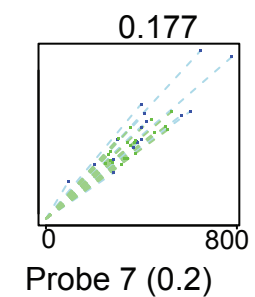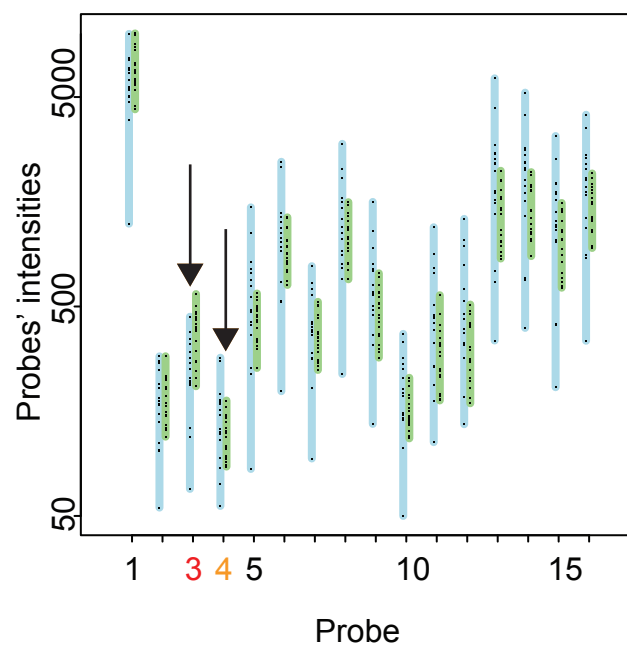

Samples from:

normal tissue  
tumour tissue

Supplement: Additional file 3 — BAD probe prevents detection of DE. Fluorescence levels of the probe set 38657_s_at for normal lung and tumor lung tissue. Each dot represents a sample. This probe set is detected as DE (alpha = 0.01) only after masking. Intensities and its correlations with some other probes of the probe set are shown for the probe 3, identified as BAD (left) and probe 4, non-BAD (right). In the middle fluorescence levels for consecutive probes. [file 1471-2105-13-56-S3.pdf]
